# EHO-85: A Multifunctional Amorphous Hydrogel for Wound Healing Containing *Olea europaea* Leaf Extract: Effects on Wound Microenvironment and Preclinical Evaluation

**DOI:** 10.3390/jcm11051229

**Published:** 2022-02-24

**Authors:** Antonio Casado-Díaz, Manuel La Torre, Feliciano Priego-Capote, José Verdú-Soriano, José Luis Lázaro-Martínez, Leocadio Rodríguez-Mañas, Miriam Berenguer Pérez, Isaac Tunez

**Affiliations:** 1 Clinical Management Unit of Endocrinology and Nutrition, Reina Sofía University Hospital, University of Córdoba, 14004 Córdoba, Spain; 2 Consortium for Biomedical Research in Frailty & Healthy Ageing, CIBERFES, Carlos III Institute of Health, 28029 Madrid, Spain; q72prcaf@uco.es (F.P.-C.); leocadio.rodriguez@salud.madrid.org (L.R.-M.); 3 Maimónides Institute of Biomedical Research (IMIBIC), Reina Sofía University Hospital, University of Córdoba, 14004 Córdoba, Spain; mlatorre@uco.es (M.L.T.); isaac.tunez@juntadeandalucia.es (I.T.); 4 Department of Biochemistry and Molecular Biology, Faculty of Medicine and Nursing, University of Córdoba, 14004 Córdoba, Spain; 5 Department of Analytical Chemistry, Institute of Nanochemistry, University of Córdoba, 14071 Córdoba, Spain; 6 Department of Community Nursing, Preventive Medicine, Public Health and History of Science, Faculty of Health Sciences, University of Alicante, 03690 Alicante, Spain; miriam.berenguer@ua.es; 7 Diabetic Foot Unit, University Podiatry Clinic, Complutense University of Madrid, 28040 Madrid, Spain; diabetes@ucm.es; 8 Department of Geriatrics, Hospital Universitario de Getafe, 28905 Madrid, Spain

**Keywords:** hydrogel, EHO-85, *Olea europaea* leaf extract, moistening, ROS, antioxidant, pH, wound healing, preclinical

## Abstract

The prevalence of chronic wounds is increasing due to the population aging and associated pathologies, such as diabetes. These ulcers have an important socio-economic impact. Thus, it is necessary to design new products for their treatment with an adequate cost/effectiveness ratio. Among these products are amorphous hydrogels. Their composition can be manipulated to provide a favorable environment for ulcer healing. The aim of this study was to evaluate a novel multifunctional amorphous hydrogel (EHO-85), containing *Olea europaea* leaf extract, designed to enhance the wound healing process. For this purpose, its moistening ability, antioxidant capacity, effect on pH in the wound bed of experimental rats, and the effect on wound healing in a murine model of impaired wound healing were assessed. EHO-85 proved to be a remarkable moisturizer and its application in a rat skin wound model showed a significant antioxidant effect, decreasing lipid peroxidation in the wound bed. EHO-85 also decreased the pH of the ulcer bed from day 1. In addition, in mice (BKS. Cg-m +/+ Leprdb) EHO-85 treatment showed superior wound healing rates compared to hydrocolloid dressing. In conclusion, EHO-85 can speed up the closure of hard-to-heal wounds due to its multifunctional properties that are able to modulate the wound microenvironment, mainly through its remarkable effect on reactive oxygen species, pH, and moistening regulation.

## 1. Introduction

Skin wounds constitute a major expense for public health systems. Their high rates of chronification, recurrence, and morbidity are associated with a significant burden on health-related quality of life, resulting in higher economic costs, both personal and social and a challenge for public health systems worldwide [1,2,3,4]. The prevalence of skin wounds and their tendency to become chronic is expected to increase due to progressive population ageing and the rising prevalence of obesity, diabetes, and cardiovascular diseases [4]. Therefore, wound management is a significant and growing issue worldwide, so the design of new products capable of favoring the healing process and shortening its duration is of great importance [5]. 

Wound healing is a complex process characterized by a series of four stages that do not always occur sequentially but rather overlap: hemostasis, inflammation, proliferation, and remodeling [6]. Meanwhile, a wide variety of growth factors, cytokines, and hormones, in combination with the preservation of adequate levels of reactive oxygen species (ROS), pH, and moisture in the wound bed, are involved in this biological process [7]. Promoting wound healing is a major therapeutic challenge. Considering observations from the mechanisms involved in the development of ulcers and its chronification, it is now widely accepted that an effective dressing within the ulcer healing strategy should possess the following properties [7,8]: (1) provide a platform that generates a supportive scaffold in the ulcer bed; (2) provide and maintain a balanced moist environment; (3) absorb exudate and bacteria and, thus, protect against secondary infections; (4) promote wound debridement; (5) maintain an adequate, discreetly acidic pH; (6) control the excess of free radicals through antioxidant activity; (7) decrease or eliminate trauma to the damaged area; (8) not possess toxic, irritant, or allergenic properties; (9) be acceptable/accepted by the patient; and (10) be cost-effective. Therefore, the treatment and management of wound healing must be approached holistically, including each and every aspect involved in the natural healing process [9].

Amorphous hydrogels are considered ideal candidates for dressings due to their excellent biocompatibility. In addition, their three-dimensional structure can provide an adequate platform to generate a supportive structure in the wound bed, creating a barrier for mechanical protection and thermal insulation. Therefore, this composition allows cell infiltration, diffusion of nutrients, metabolites and water-soluble molecules, and simple gas exchange through a highly permeable platform [10]. Hydrogels also possess the ability to donate moisture to the wound bed, as well as to absorb a certain amount of the exudate and debris contained in the wound within their reticulate structure. This process facilitates autolytic wound debridement, thereby providing the moisture balance at the wound surface required for wound repair, which overcomes the shortcomings of traditional dressings [11]. These are commonly accepted qualities shared by almost all widely used amorphous hydrogels today. Because of all these characteristics, hydrogels can be used in all types of ulcers (venous, pressure, diabetic, surgical wounds, burns, etc.) and at any stage of the ulcer healing process, from dry necrotic wounds to rehydrating the wound bed and providing a moist wound healing environment. In addition, hydrogels can also be used on granulation tissue and in the re-epithelialization phase, where new connective tissue and new skin form, respectively [12,13]. Subsequently, multifunctional hydrogels with good biocompatibility are currently considered the baseline option for wound healing, since they can achieve multistage and multifunctional combination therapy [14,15,16].

It is widely proven that moist occlusive or semi-occlusive environments for wound healing double wound healing rates when compared to dry ones [17]. Specifically, moist wound healing (MWH) consists of keeping the wound bed isolated from external factors by providing a semi-occlusive and moist environment, with the wound exudate in permanent contact with the wound. The moist environment also contributes to maintaining a slightly acidic pH, which helps to produce a low-oxygen surface tension that stimulates angiogenesis and, thus, accelerates the wound healing process [18]. Moreover, once the moist environment has been created, wound exudate serves as a transport vehicle for a variety of bioactive molecules, such as enzymes, growth factors, and hormones, which naturally increase fibroblast, keratinocyte, and endothelial cell growth and division, as tested both in vitro [19], and in vivo [20]. In addition, maintaining an appropriate temperature and humidity favors pro-healing chemical reactions, cell migration, and debridement of devitalized tissue [21]. All the properties provided by MWH are the same as those inherently possessed by acute wounds in the early stages of their natural evolution [18].

There is evidence from in vivo and in vitro studies of the predominant role of ROS in the multiple stages of pathogenesis leading to the failure of wounds to heal [22,23]. The modulation of ROS to avoid excessive and sustained increases in oxidative stress over time is, therefore, a critical aspect during the wound healing process since it can contribute significantly to its acceleration [16,24,25]. Some authors have expressly proposed the urgency of developing hydrogel dressings with antioxidant properties [16]. Among non-enzymatic antioxidants, polyphenols, such as those contained in *Olea europaea* leaf extract (OELE), have been extensively assessed due to their outstanding antioxidant properties [26,27,28]. In this sense, OELE and oleuropein, the most abundant polyphenol in OELE, have shown the ability to promote wound healing in in vivo models [29,30,31,32]. In part, this capacity is mediated by their antioxidant properties [31,32].

The wound healing process is strongly associated with changes in the wound bed pH, which directly and indirectly influences all physiological events involved in the wound healing process [33]. The pH of healthy skin is acidic on the surface, but when a wound occurs, deeper layers are exposed, creating an alkaline microenvironment (approximate pH of 7.4), which hinders and slows down the healing process and favors colonization by pathogens.

In an adequate progression of wound healing, the wound environment moves from an alkaline to a neutral pH and then to an acidic pH as healing begins [34]. It has been broadly observed that wound healing progression decreases when the pH is raised to an alkaline condition: both acute and chronic wounds with a highly alkaline pH have a lower healing rate compared to wounds with a more neutral pH [34,35,36,37]. Therefore, induction of a mildly acidic environment in the wound can be beneficial in several respects: the acidic pH of a wound surface plays an important role in wound healing, as it contributes to enhance antimicrobial activity, oxygen release, angiogenesis, protease activity, and bacterial toxicity [38]. Hence, the pH value in wounds is a dynamic factor of great importance that can be rapidly modified by therapeutic interventions. Wound pH control and lowering have been proven as key therapeutic targets to enhance wound healing in a recent literature review [38].

Taking into account the importance of creating a favorable environment characterized by (i) a suitable moist environment for skin wound healing, (ii) a reduction of excess free radicals, and (iii) a discreetly acidic pH in the wound bed, a novel multifunctional amorphous hydrogel containing OELE, was designed (EHO-85). Thus, the aim of the present study was to assess the aforementioned properties of the EHO-85 hydrogel. For this purpose, we investigated: (i) the moistening ability of EHO-85 in comparison to other amorphous hydrogels used in general wound care practice; (ii) the antioxidant (scavenger) capacity of the EHO-85 hydrogel in the wound bed of experimental animals, assessed as lipid peroxidation; and (iii) the effect of EHO-85 on wound bed pH in an animal model. Finally, to determine the ability of EHO-85 hydrogel to accelerate the healing of skin ulcers, (iv) we assessed and compared the effect of EHO-85 hydrogel on wound healing in a murine model of impaired wound healing.

## 2. Materials and Methods

### 2.1. Amorphous Hydrogel Containing Olea Europaea Leaf Extract (EHO-85)

EHO-85 is composed of purified water, OELE, Fucocert^®^, glycerin, Carbopol 980^®^, trieathanolamine, disodic ethylenediaminetetraacetic acid (EDTA), and Geogard ultra^®^ [39]. It was formulated with a slightly acidic pH (5–5.5). The characteristic component of EHO-85 is OELE, which was prepared from Andalusian olive trees (Spain) by Ferrer HealthTech (Murcia, Spain). OELE was added at a concentration of 0.1%, since it was the lowest concentration at which complete healing was achieved in a previous experiment in db/db mice [39]. The cross-linked acrylic acid polymer Carbopol 980^®^ was selected due to its easy and fast dispersion properties. Additionally, it provides good rheological properties and has been widely used for more than forty years in the pharmaceutical industry for human skin care [40]. Triethanolamine was employed as a gelling neutralizing agent of the polymer and to form the gel network [41]. Geogard Ultra^®^ (Gluconolactone and sodium benzoate] was added to the formulation to avoid microbiological contamination. Two agents commonly used for skin care, glycerin and Fucocert^®^, were also included. The first one is commonly reported as key component for moisturizing, repair, and elasticity [42,43]. Fucocert^®^ consists of three sequential sugars (L-fucose, D-galactose, and acid galacturonic), which have moisturizing, soft touch, and self-emulsifier properties, additionally contributing to set a protective film over the wound [44]. In addition, EDTA was incorporated into the formulation for its properties as an antimicrobial and antibiofilm agent [45].

### 2.2. Moistening Ability of EHO-85 Compared to Other Amorphous Hydrogels

The ability of the EHO-85 and other amorphous hydrogels to donate liquid was determined at the Department of Analytical Chemistry of the University of Cordoba (Spain) using the standardized method proposed in standard BS EN 13726-1:2002 [46]. According to that method, 10 ± 0.1 g samples of the test materials were placed onto the surface of a series of 10 ± 0.1 g plugs of gelatin (35%). The gelatin was contained within the barrel of 50 mL syringes from which the closed (nozzle) ends were removed to form smooth-sided cylinders. Once the test materials were in place, the open ends of the cylinders were sealed with an impermeable cover. Following incubation of the sealed syringes for 48 h at 25 °C, the test materials were gently removed from the plugs, which were then re-weighed. From these results, the percentage changes (losses) in the weight of each hydrogel sample were calculated. The classification of the hydrogels according to their fluid donation capacity was preset by the standard, ranging from A to E (0–5% and 20–25% loss in gel weight, respectively) (Table 1).

Samples of frequently used amorphous hydrogels were assessed in order to compare their liquid donation ability with that of EHO-85: Askina^®^ Gel (B Braun Medical, Rubí, Spain), batch code (BC): 400262; Normlgel^®^ (Mölnlycke, Alcobendas, Spain), BC: 13362978; Purilon^®^ Gel (Coloplast, Madrid, Spain), BC: 238572; Intrasite Gel^®^ (Smith & Nephew, Sant Joan Despí, Spain), BC: 131961; Nu-Gel^®^ Hydrogel (Systagenix, Madrid, Spain), BC: 2078866; and EHO-85 hydrogel, BC: 85.1.

### 2.3. Rat Wound Model for the Study of the Effect of EHO-85 on Lipid Peroxidation and pH in the Wound Bed

Fourteen-week-old female Wistar rats were used for the assays. The animals were kept at 20–23 °C in a 12 h light/12 h dark cycle, with food (Purine^®^, Barcelona, Spain) and water ad libitum. All experimental procedures were reviewed and approved by the Animal Research Ethics Committee of the University of Cordoba, Spain, and the Institutional Animal Care Committee (ref. 16/10/2017/138). All procedures were performed according to the Guide for the Care and Use of Laboratory Animals and following European and Spanish animal welfare laws.

Animals were under general anesthesia, with xylazine (5 mg/kg) and ketamine (80 mg/kg) administered intraperitoneally. Their dorsum was shaved and, using surgical scissors and forceps, two excisions of 12 mm in diameter each were made in each animal, covering the entire thickness of the skin up to the fascia. To prevent the animals from touching the wounds and removing the dressings that would later be applied, ad hoc tape collars and belts were made and placed around the neck and abdomen, though allowing animals to move around and feed properly.

### 2.4. Evaluation of the Antioxidant (Scavenger) Capacity of Hydrogel Containing EHO-85 in the Wound Bed of the Rat Wound Model, Assessed As Lipid Peroxidation and Reduced/Oxidized Glutathione Ratio

To assess the scavenger ability of the EHO-85 hydrogel in vivo, the rat model described in the Section 2.3 was used.

The animals were randomly assigned to two groups. Group 1 (*n* = 8) acted as a control. It was treated with the EHO-85 hydrogel without OELE plus an occlusive dressing. Group 2 (*n* = 8) was treated with the EHO-85 hydrogel and the same occlusive dressing. To evaluate the oxidative stress generated in the wounds during the initial phase of inflammation and its hypothetical subsequent decline, four animals from each group were sacrificed at 48 and 96 h after wounding. The wounds were excised and frozen in liquid nitrogen. Extracts were obtained from the samples and lipid peroxidation was determined in both groups by means of the LPO-586 kit^®^ (Oxis International, Portland, OR. USA). This assay uses a chromogenic reagent that reacts with the products of lipid peroxidation, malondialdehyde (MDA) and 4-hydroxyalkenals (HAE) (at 45 °C), producing a stable chromophore with a maximum absorbance peak at 586 nm. The obtained values were normalized to the amount of protein in the samples, as determined by the conventional Bradford method and expressed as nMol (MDA + 4HDA)/mg protein.

The ratio of reduced and oxidized glutathione (GSH/GSSG) was determined as an antioxidant marker in the tissue. For this purpose, the GSH/GSSG-412TM kit (Oxis International) was used according to its instructions. The concentrations of total and oxidized glutathione were determined spectrophotometrically at 412 nm and normalized with the samples’ protein concentrations. The GSH/GSSG ratio was obtained from the collected data.

### 2.5. Effect of EHO-85 on Wound pH in an Animal Model

The study was carried out on the rat wound model described in Section 2.3. Animals were randomly distributed in two groups: control (no treatment) (*n* = 4) and experimental (treatment every 48 h with EHO-85 hydrogel) (*n* = 4).

The duration of the study was 7 days. On day 0 (start of the experiment), the pH of each wound was measured using a flat-tipped electrode for surface pH measurement (Hanna Instruments; Eibar, Spain). Next, the EHO-85 hydrogel was applied to the experimental group. Immediately afterwards, the wounds of both groups were covered with a transparent plastic dressing (Tegarderm film^®^, 3M, Madrid, Spain). The pH measurement was repeated every two days, coinciding with the application of the EHO-85 hydrogel (days 1, 3, 5, and 7 after the wounds were made). For this purpose, dressings were previously removed, and the wounds were washed with physiological saline (NaCl 0.9%, B Braun Medical, Rubí, Spain).

### 2.6. Effect of EHO-85 on Wound Healing in a Murine Model of Impaired Wound Healing (BKS. Cg-m +/+ Leprdb)

A 10-12-week-old female db/db diabetic mouse model (BKS.Cg-m +/+ Leprdb, Laboratoires Janvier, 4105-Saint Berthevin, France) was chosen for being a well-known model of impaired wound healing [47,48]. The test was conducted according to Directive 2010/63/UE and Standard ISO 10993-2 and following the OECD Principles of Good Laboratory Practice. The animals had free access to a dry, pelleted standard rodent diet (A04 Safe Maintenance Diet for rodents, Panlab S.L., Barcelona, Spain) and allowed ad libitum access to drinking water. Prior to the start of the study, and once the quarantine was over, both flanks of each animal were shaved. On day 0 of the study, the animals were anesthetized with ketamine (100 mg/kg) and xylazine (10 mg/kg) administered intraperitoneally. Then, two excisional wounds of 10 mm diameter were made on each dorsal side of the mice. The wounds were performed under aseptic conditions using surgical instruments. After the wounds were made, digital photographs of the wounds were taken.

The animals were randomly assigned to four treatments groups, with four animals in each and were housed in one cage per group, according to Directive 2010/63/UE. The treatment groups were as follows: group A (EHO-85 hydrogel), a fine layer of approximately 20 μL of EHO-85 was applied directly into the wounds; group B (hydrocolloid dressing, positive control), wounds were treated with a hydrocolloid dressing (Hydrocoll^®^, Hartmann. Barcelona. Spain); group C (standard amorphous hydrogel), the wounds were treated with a 20 μL layer of the hydrogel formulated without OELE; and group D (Control), wounds did not receive any specific treatment. To prevent the animals from touching the wounds, post-surgical dressings were applied.

Treatments were applied every other day until day 14 or until complete wound closure if this occurred earlier (days 2, 4, 6, 8, 10, 12, and 14). Wound healing was monitored every two days throughout the 14 days trial by digital camera photography. Ulcer surface reduction was analyzed by ImageJ software 1.53f51 from the National Institutes of Health (NIH; Bethesda, MD, USA).

### 2.7. Statistical Analysis

Variables were expressed as means and standard deviations (SD). Comparisons between groups regarding wound healing activity were analyzed with the analysis of variance (ANOVA) and Dunnett’s post-hoc test. Student’s *t*-test was used to analyze differences between two groups. Statistical significance was established with *p* ≤ 0.05. All statistical procedures were carried out with GraphPad Prism 8.0 program from GraphPad Software (San Diego, CA, USA).

## 3. Results

### 3.1. Moistening Capacity of the EHO-85 Hydrogel Compared to Other Amorphous Hydrogels

The results obtained in the fluid donation test are presented in Table 2, in which the gels are classified according to the BS EN 13726-1 norm standards (Table 1). The results are shown as means and standard deviations of the mean. Based upon the classification stated by Test Method BS EN 13726 (40), the product under study (EHO-85) lost, on average, 15.2% of gel weight. Consequently, it was classified as a Type-D hydrogel.

Therefore, the EHO-85 hydrogel possessed a very significant moistening capacity. Its liquid donation ability was superior to the average of the amorphous hydrogels that constitute the current state of the technique.

### 3.2. Evaluation of the Antioxidant (Scavenger) Capacity of the EHO-85 Hydrogel in the Wound Bed of Experimental Animals, Assessed As Lipid Peroxidation and the GSH/GSSG Ratio

Oxidative stress in the animal wounds, measured as lipid peroxidation, increased significantly from 48 to 96 h post-wounding, as was expected to occur during the initial inflammation phase. However, the group of animals treated with the EHO-85 hydrogel containing 0.1% OELE showed a significant scavenger effect, significantly decreasing lipid peroxidation in the wound bed at both 48 and 96 h compared to control (*p* < 0.001) (Table 3).

As an antioxidant marker, the GSH/GSSG ratio was quantified. Under oxidative stress conditions, GSH is oxidized to GSSG by glutathione peroxidase. The oxidized form is recycled to GSH by glutathione reductase. Under oxidative stress conditions, the GSSG form accumulates in the tissue and the GSH/GSSG ratio decreases. At 48 h of treatment, no significant differences in the GSH/GSSG ratio were observed in wounds treated with EHO-85 compared to those treated with the hydrogel without OELE. However, at 96 h, in wounds treated with EHO-85 containing OELE, the GSH/GSSG ratio was significantly higher (Table 4). These results indicate that wounds treated with EHO-85 possess a higher antioxidant capacity.

### 3.3. Effect of EHO-85 on Wound Bed pH in an Animal Model

**Figure 1:**
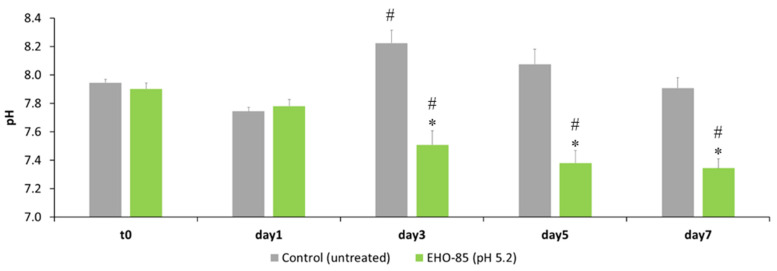
Effect of EHO-85 treatment on wound pH in Wistar rats. The results are shown as means ± standard deviations. * *p* < 0.05 compared to control, # *p* < 0.05 compared to t = 0 days (before treatment).

As can be appreciated in Figure 1, the application of the hydrogel under study (EHO-85) in the wound of rats induced a slight and sustained reduction in the pH of the ulcer bed from day 1 onwards, as opposed to the untreated animals in which pH downregulation was not achieved. The average difference in pH reduction between both groups can be estimated at around 0.6 units on days 3, 5, and 7 (end of study).

### 3.4. Effect of EHO-85 Amorphous Hydrogel with Antioxidant Properties on Wound Healing in a Murine Model of Impaired Wound Healing (BKS. Cg-m +/+ Leprdb)

**Figure 2:**
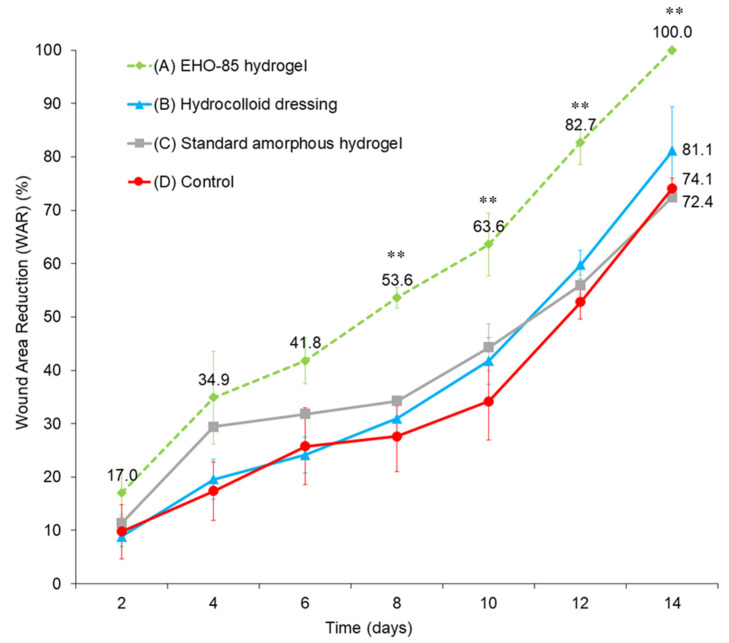
Wound area reduction. The results are shown as means of the animals in each group (*n* = 4) ± standard deviations. ** *p* < 0.01 compared to control group D (untreated).

The EHO-85 hydrogel showed superior wound healing rates compared to the reference treatment (hydrocolloid dressing). As opposed to the animals treated with hydrocolloids, those in which the EHO-85 hydrogel was applied reached significant differences in the percentage of wound healing versus the untreated group (*p* < 0.01 from day 8 onwards). Interestingly, the EHO-85 hydrogel showed a significantly higher wound healing ability (*p* < 0.01) than the standard amorphous hydrogel formulated without its novel antioxidant and acidifying properties (Figure 2).

Regarding the safety of the product, no signs of allergy, irritation, or sensitization were observed in any of the animals of the study. The tissue surrounding the wound did not suffer signs of additional deterioration (erythema, oedema, eczema, maceration, etc.).

## 4. Discussion

Due to the increasing challenge of the problem posed by skin ulcers, advances in the knowledge of their approach are required, along with new dressings with better clinical results. Wounds in the usual practice are currently treated by focusing on their signs and symptoms (level of exudate, wound bed tissue, and presence of infection, mainly), with little attention directed to other underlying phenomena occurring in the ulcer microenvironment that may cause their chronification, such as tissue hypoxia, inadequate moisture, persistent inflammation, defects in the extracellular matrix, raised levels of alkalinization and ROS, or the presence of metalloproteases [49,50]. In this context, multifunctional dressings able to modulate the wound microenvironment throughout the entire healing process, besides acting as physical protective barriers against infection, are currently of great interest.

In line with the above, a new hydrogel (EHO-85) was designed with the aim of modulating the wound microenvironment, combining the moisturizing and insulating capacity inherent to any amorphous hydrogel with the ability to act as a scavenger of excess ROS from the ulcer microenvironment and to lower the pH of the lesion. The set of preclinical tests included in the present study have confirmed the multifunctional mechanism of action attributed to the novel EHO-85 hydrogel. Additionally, it has permitted the assessment of its capacity to accelerate the wound healing process in an in vivo model as a necessary prior step to the subsequent randomized clinical trial.

### 4.1. Moist Wound Healing (MWH): Moisturizing Effect on Wound Healing

It is widely accepted that generating a warm and moist wound environment promotes wound healing [51], so maintaining moist wound conditions is a crucial wound management challenge [49,52]. Therefore, the EHO-85 hydrogel was endowed in the right proportions with glycerin and Fucocert^®^, highly moisturizing components that provide a significant liquid donation ability, classified as Type D according to standard BS EN 13726 [46], and superior to the average of the tested amorphous hydrogels that constitute the current state of the technique. Moreover, its design allows EHO-85 hydrogel to act as a permeable 3D matrix that can adapt to the wound bed configuration, thus overcoming some of the deficiencies of traditional wound dressings.

The currently available evidence shows that a warm and moist wound environment favors wound healing [53] in such a way that hydrogel dressings (able to keep the wound moist and absorb large amounts of exudate [54]) are currently considered a reference among chronic wound treatment dressings [55].

### 4.2. Oxidative Stress Reduction and Wound Healing

In the normal wound healing process, throughout the initial inflammatory phase, the concentration of neutrophils and macrophages in the ulcer bed generates large amounts of reactive oxygen species, such as HO_2_-, HO-, and O_2_-. ROS maintained at low concentrations play a favorable role in wound healing, participating in the defense against micro-organisms and infections, as well as in signal transduction for re-epithelialization, cell proliferation, and cell repair [24,25]. However, if the inflammatory phase is not resolved in a timely manner and is prolonged, a large accumulation of ROS occurs, which exceeds the antioxidant capacity of the system. This is the case of chronic wounds or wounds in the course of chronification. Excessive ROS in wounds damage cell membranes and macromolecules, such as extracellular proteins, lipids, and deoxyribonucleic acid (DNA). This has a significant harmful effect on the cellular and vascular processes involved in wound healing [56] and contributes to the activation of complex pro-inflammatory signaling pathways, thus hindering the transition of the wound from the inflammatory to the proliferative phase [24,25,56]. Therefore, decreasing ROS levels through the use of an antioxidant agent may decrease inflammation and favor the progression of the healing process. The ability of EHO-85 to easily release antioxidant agents into the wound is due to the inclusion of OELE, which permits the maintenance of an appropriate redox status. Thus, through the mechanism described above, EHO-85 may favor the creation of a favorable microenvironment for the promotion of the wound healing process, especially in hard-to-heal wounds or wounds in the chronification process [50].

A relevant target to accelerate wound healing involves a correct regulation of the redox indicators associated with the healing process [16,57], such as lipid peroxidation, which is a standardized measure of the cell damage due to oxidative stress. The interaction of oxygen free radicals with molecules of a lipid nature produces new radicals, such as superoxide, hydroxyl, and lipoid peroxides, which, in turn, can interact with biological systems in a clearly cytotoxic way. Flavonoids, phenols, and oleuropeosides, the main phenolic compounds present in OELE, have been shown to possess a significant antioxidant activity towards these radicals. This activity is mainly based on the redox properties of their phenolic hydroxyl groups and the structural relationships between different parts of their chemical structure [26]. Oleuropein is the most prominent phenolic compound in OELE, usually followed by hydroxytyrosol, oleuropein, aglycone, and tyrosol [27]. Furthermore, OELE phenolic compounds have a synergistic effect on antioxidant capacity when they are together in the extract compared to their individual effects [58].

A recent study by our group showed that the presence of OELE decreased lipid peroxidation and ROS production, while it increased the survival of dermal fibroblast and keratinocyte cultures exposed to oxidative stress conditions in vitro [39]. Since these cells play a key role in skin wound healing, these results support those obtained in the present study, in which the presence of the OELE in the EHO-85 hydrogel reduced by half the lipid peroxidation in the wound bed in an in vivo model. This capacity of the novel antioxidant hydrogel to act as an ROS scavenger in a rapid (48 h) and steady (96 h) way, controlling excess ROS early, may significantly enhance the wound repair process [57]. Moreover, the presence of OELE in the hydrogel also resulted in a higher GSH/GSSG ratio in the wounds after 96 h of treatment when compared to wounds treated with the hydrogel alone. This means that OELE, in addition to reducing oxidative damage expressed as lipid peroxidation, allows a greater intrinsic antioxidant capacity to be maintained in the tissue. These results coincide with those described in other studies. For example, OELE treatment in rats with carbon-tetrachloride-induced liver damage decreased lipid peroxidation in the tissue and increased GSH concentration [59]. Moreover, in rats treated with Doxorubicin, a drug used in chemotherapy that produces important secondary effects through the induction of oxidative stress, treatment with OELE decreased lipid peroxidation and increased GSH levels in different organs [60]. Therefore, our results, together with these data, confirm the power of OELE as an ROS scavenger able to decrease oxidative stress and maintain the functionality and regenerative capacity of different organs.

It has been shown in several experimental mouse models that the application of OELE or oleuropein to wounds contributes to significantly accelerate healing of skin ulcers due to its high antioxidant capacity [29,30,31,32,39]. Our results support the findings of other studies that have also evaluated the effect of hydrogels containing plant extracts with high antioxidant capacitoes. In these studies, the presence of different plant extracts in the hydrogels favored the skin wound healing in animal models, in part due to their antioxidative effect [61,62,63].

### 4.3. pH Regulation and Wound Healing

Another key mechanism to modulate the wound microenvironment is the regulation of its pH. The administration of the EHO-85 hydrogel (pH 5.0–5.5) induced a slightly acidic environment on the wound bed as early as the first day after its application. This pH downregulation was maintained over time (Figure 1). This is beneficial for ulcer healing because it contributes to enhance the various reparative processes in the wound by increasing the physiological activity of macrophages and fibroblasts [64] and modifies matrix metalloproteinases [65]. Furthermore, the acidic pH facilitates the release of oxygen into the wound. This could help to maintain the slightly acidic pH (5.5–6.6) generated previously though EHO-85 application and produces a low surface oxygen tension that stimulates angiogenesis, thus speeding up the wound healing process [18]. A reduction in wound pH, such as that generated by the EHO-85 hydrogel, could directly influence tissue oxygen release in the wound. Interestingly, increases in oxygen release on wounds caused by wound tissue acidification are exponential. Therefore, a 0.9 unit change in pH would result in a 5-fold increase in oxygen release. Similarly, a pH decrease of 0.6 units, as induced by the EHO-85 hydrogel (Figure 2), would release almost 50% more oxygen, which would contribute to significatively improving the epithelialization process due to better oxygenation speeding up the wound healing process [66]. This fact explains the efficacy of hyperbaric oxygen therapy. The probability of ulcer healing increases if the tissue oxygen tension (pO2) is higher than 40 mm Hg. Conversely, it decreases when it is below 20 mm Hg [38,67]. In addition, an acidic pH improves the natural barrier function and helps to counteract the potential microbial colonization of bacteria [68] or *Candida albicans* [69], which require alkaline pH values in the wound bed, while their growth is inhibited by more acidic pH values.

The benefit of obtaining an acidic environment to improve the natural history of ulcer healing has been supported by several studies and observational clinical studies in patients. These studies show that both chronic and acute wounds with a highly alkaline pH have higher healing rates when treated with an acidic pH [34,35,70].

### 4.4. Wound Healing Promotion In Vivo

In order to corroborate our hypothesis that the novel EHO-85 hydrogel could constitute a suitable contribution to the promotion of wounds, an in vivo wound healing test was carried out using a murine model of impaired wound healing (BKS. Cg-m +/+ Leprdb). This mouse species was selected since diabetes has been shown to affect many components of wound healing, including impaired blood flow in skin wounds, as well as decreased neutrophil anti-microbial capacity, abnormal expression of chemokines, and a reduction in certain growth factors essential for healing. Given these analogies with human chronic wound healing, it is a useful model for the study of wound healing mechanisms and for the evaluation of new therapeutic modalities [47].

The topical application of the EHO-85 hydrogel accelerated the healing process of ulcers in diabetic mice. It is important that treatment of diabetic animal wounds with EHO-85 accelerates the healing process in short periods of time. In this study, wounds in diabetic animals treated with topically applied EHO-85 hydrogel healed faster as early as day 4. As opposed to the reference treatment (hydrocolloid dressing), the EHO-85 hydrogel was capable of reaching significant differences in the percentage of wound closure versus negative controls (*p* < 0.01) and did so from day 8 onwards. Interestingly, the EHO-85 hydrogel showed a significantly higher wound healing ability (*p* < 0.01) than the same amorphous hydrogel formulated with the same basis but without its characteristic antioxidant and acidifying properties. This would confirm the relevance of the holistic approach to wound management made possible by the EHO-85 dressing.

## 5. Conclusions

EHO-85, a hydrogel containing *Olea europaea* leaf extract with an innovative design possesses and novel multifunctional properties, is able to modulate the wound microenvironment, mainly through its antioxidant effect and pH and moistening regulation. These mechanisms of action cooperate in a balanced way with the natural physiological processes involved in wound healing, thus further improving the therapeutic effect of current dressings and accelerating the healing of hard-to-heal wounds.

## Figures and Tables

**Table 1:** Liquid donation capacity rating of the hydrogels according to standard BS EN 13726-1:2002.

Fluid Affinity (Donation) (%) Loss in Gel Weight	Type
0–5	A
>5–10	B
>10–15	C
>15–20	D
>20–25	E

**Table 2:** Liquid donation capacity of the hydrogels (gelatine 35%), expressed as means ± standard deviations in the samples (*n* = 4).

Hydrogel	(%) Average Decrease of Gel Weight	Type
Askina^®^ Gel	19.0 ± 2.0	D
Normlgel^®^	13.0 ± 3.0	C
Purilon^®^ Gel	12.5 ± 0.3	C
Intrasite^®^ Gel	6.0 ± 2.0	B
Nu-Gel^®^	9.0 ± 3.0	B
EHO-85	15.2 ± 0.9	D

**Table 3:** Effect on oxidative stress in the wound of animals treated with EHO-85 hydrogel containing OELE vs. EHO-85 hydrogel formulation without extract, measured by the effect on lipid peroxidation. Results are shown as the mean µM (MDA + 4HDA) /mg protein ± standard deviation of four animals per group.

	µM (MDA + 4HDA)/mg Protein		
Treatment	48 h	96 h	
EHO-85 without 0.1% OELE	0.0097 ± 0.0005	0.0134 ± 0.0008	*p* < 0.001
EHO-85 hydrogel	0.0052 ± 0.0003	0.0073 ± 0.0004	*p* < 0.001
	*p* < 0.001	*p* < 0.001	

**Table 4:** Effect of the presence of OELE on the GSH/GSSG ratio obtained from the wounds of animals treated for 48 and 96 h with EHO-85 hydrogel containing OELE vs. the EHO-85 hydrogel formulation without extract.

	GSH/GSSG		
Treatment	48 h	96 h	
EHO-85 without 0.1% OELE	0.382 ± 0.093	0.743 ± 0.050	*p* < 0.05
EHO-85	0.346 ± 0.034	1.195 ± 0.174	*p* < 0.05
	ns	*p* < 0.05	

## Data Availability

The data presented in this study are available on request from the corresponding author.
